# Supplementary material for: Somatosensory-visual effects in visual biological motion perception
Source: PLoS One. 2020 Jun 11;15(6):e0234026. doi: 10.1371/journal.pone.0234026 (PMC7289375; doi:10.1371/journal.pone.0234026)
Supplement: Attachment — Submitted filename: Response to Reviewers.docx [file pone.0234026.s001.docx]

Pierre Progin	September 30th, 2019

Laboratory of Cognitive Neuroscience

Swiss Federal Institute of Technology

Campus Biotech H4

Chemin des Mines 9

CH-1202 Genève

Switzerland

**RESPONSE TO REVIEWERS**

Dear Dr. Pavlova,

Thank you for giving us the opportunity to submit a revised draft of our manuscript titled “Somatosensory-visual effects in visual biological motion perception” to *PlosOne*. We appreciate the time and effort that you and the reviewers have dedicated to providing your valuable feedback on our manuscript. We are grateful to the reviewers for their insightful comments on our paper. We have been able to incorporate changes to reflect most of the suggestions provided by the reviewers. We have highlighted the changes within the manuscript.

Here is a point-by-point response to the reviewers’ comments and concerns. Please, find below the reviewers’ comments repeated in italics and our responses inserted after each comment.

We look forward to hearing from you in due time regarding our submission and to respond to any further questions and comments you may have.

Sincerely,

Pierre Progin and co-authors

**Comments from Reviewer #1:** *Authors depeen somatosensory effect, in particular tactile foot stimulation, on BM visual-perception, basing on evidence of brain areas integrating auditory and visual signals. I found the study interesting and the purpose of the research relevant. I would suggest some clarifications in order to make the manuscript clearer:*

*ABSTRACT:*

*Even though it explains and justifies the aim of the research, it does not give space to the method. I suggest adding method of the research in term of the design, number of experiments, tasks, participants....*

Response: Thank you for pointing this out. We agree with this comment. Therefore, we have added information about the methodological part of the study in the abstract:

*In two experiments, we asked healthy participants to perform a speed discrimination task on two point light walkers (PLW) presented one after the other. In the first experiment, we quantified somatosensory-visual interactions by presenting PLW together with tactile stimuli either on the participants’ forearms or feet soles. In the second experiment, we assessed the specificity of these interactions by presenting tactile stimuli either synchronously or asynchronously with upright or inverted PLW.*

*INTRODUCTION:*

*I found the introduction section well written, reporting background related to research questions. Nevertheless, I did not find a section showing literature that lead authors to investigate temporal specificity of somato-visual interactions. I would suggest integrating in introduction this part.*

Response: We agree with this point and have revised the introduction accordingly. A first reference to a seminal neuroanatomical study (Meredith 1987) was added in the contextual part of the introduction:

*Several studies have now shown that auditory information influences visual BM processing across a range of tasks (10,46–48), given that the two signals are temporally linked (49). As Meredith and Stein found in cat superior colliculus (50), multimodal neurons in STS are more responsive to coincident and colocalized multimodal stimuli compared to spatially and temporally incoherent stimuli (51).*

A reference to audio-visual integration was also added to the description of the second experiment at the end of the introduction:

*Within the audio-visual motion processing literature (49,51), cross-modal integration is optimized under conditions of temporal co-incidence. If the somato-visual BM processing effect is of a similar nature, we expected that temporally coincident (synchronous) foot stimulation (i.e. tactile cue is applied when the seen PLW touches the ground) would result in better performance than asynchronous stimulation (i.e. tactile cue is applied at other times).*

*When authors present experiment 1 conditions (ET, CT,V), while they well describe ET and CT, they do not specify baseline condition (V).*

Response: We would like to thank the reviewer for noticing this omission. We now describe the baseline condition in the introduction:

*First, the discriminability of visual BM was compared when tactile stimulation was applied to the feet (experimental tactile condition - ET) or to the forearms (body-site control tactile condition - CT), compared to a baseline condition in which no tactile stimulation was applied (only vision condition – V).*

*METHODS:*

*I would suggest including chi-square analysis to report differences in gender distribution (females:males) in both experiments. In experiment 2 7/21 females are included, that means they are 1/3 of the sample. Is there a significant difference between males and females? A growing literature is documenting a gender effect in BM perception (Sokolov et al.,Frontiers in Psychology, 2011; Krüger et al., 2013, PloS One; Pavlova et al., 2014, Cerebral Cortex...).*

Response: We agree with this and have run a binomial test to know if our groups were significantly unbalanced for gender. In Experiment 1, we had 8 females on 21 participants, which corresponded to a p-value = 0.38. In Experiment 2, we had 7 females on 21 participants, with a corresponding p-value = 0.19. Given that groups were not significantly unbalanced, and considering that our sample size is rather small, we decided not to add this factor as a covariate of interest. However, following the reviewer’s suggestion, we now discuss the relevance of gender for BM perception:

*Although our samples were not significantly unbalanced (see methods), the influence of gender on BM perception is worth considering. Indeed, female and male observers were found to judge differently whether a frontal PLW is facing toward or backward them (57), as well as emotional expressions (58, 59). Future studies will be required to assess whether cross-modal effects on motion processing depend on gender.*

*I also suggest adding more information regarding recruitment of participants:*

Response: We now mention more details about recruitment:

*Participants were recruited through printed and electronic advertisements on notice boards at various sites in the Ecole Polytechnique Fédérale de Lausanne (EPFL). They were all student at the EPFL. After contacting the experimenter, participants received the participant information sheet explaining the procedure and the goal of the study as well as the exclusion criteria (uncorrected vision deficit, somatosensory deficit).*

*How and where participants were recruited?*

Response: Participants were recruited through advertisement on notice board in the campus EPFL at Lausanne.

*What about their education level?*

Response: All participants were student at EPFL, in bachelor or master degrees.

*Which were inclusion and exclusion criteria?*

Response: Inclusion criteria were:

1. Age between 18 and 60 years.

2. Willing to refrain from drinking alcohol at the testing days and from consuming

psychoactive substances 2 weeks before testing days and for the duration of the

study.

3. Willing and capable to give informed consent for the participation in the study

after it has been thoroughly explained.

4. Absence of neurological and major physical impairment, with normal or corrected to normal sensory abilities.

5. Informed consent form was signed.

Exclusion criteria were:

1. Prior participation in a similar study that could bias the current results

2. Presence of major internal or neurological disorders.

3. Non-compliance to the instructions of the experimenter or an inappropriate behavior hindering the normal progress of the experiment.

*Are PLW stimuli balanced in males and females stimuli?*

Response: PLWs were neutral regarding gender features and all stimuli were the same (PLWs shown in a frontal view) for every participant. We now specify this point in the revised manuscript in the “Apparatus and stimuli” part:

*The PLWs were the same for all participants.*

*DISCUSSION:*

*I would suggest starting discussion with a resume of the research question and experiment design.*

Response: We agree with this suggestion and have modified the discussion accordingly:

*The primary aim of these experiments was to investigate possible effects of somatosensory cues on the perception of visually defined biological motion. We designed Experiment 1 to compare the discriminability of visual BM when tactile stimulation was applied to the participants’ feet (ET) or forearms (CT), or when no tactile stimulation was applied (V). Experiment 2 was aimed to further assess the temporal specificity of somatosensory-visual interactions uncovered in Experiment 1, and to test whether they were specific to BM processing.*

**Comments from Reviewer #2:** *This paper aims to examine the somatosensory effects in visual biological motion perception. For this, the authors propose two studies where they used a two-interval forced choice paradigm and tested the discrimination of point-lights walkers (PLW) speed in the presence of tactile cues. More precisely, the first experiment compares the performances in speed discrimination of PLW when the visual presentation is associated or not or incongruent tactile cues. The second experiment tests the role of time synchrony and recognition of the global motion in the tactile effects. Globally, the analyses show that the speed discrimination of PLW is improved when the visual stimulation is associated with congruent tactile cue (stimulation on the feet) but this effect is only observed when the tactile stimulation is synchronous with the visual stimuli. Interestingly, this effect does not rely with the global recognition of PLW because it persists even the PLW are presented upside-down. The authors interpret their results as somatosensory-visual interactions in BM perception with particular reference to the human mirror neuron system and multisensory mechanisms in action perception.*

*Globally, I find the study timely and very interesting. Introduction is well documented, and methods and results are clear. However, I have some suggestions to improve the manuscript.*

Response: We would like to thank the reviewer for noting the quality of our work.

*INTRODUCTION:*

*I find the introduction clear and well documented even the authors could cite more recent studies about PLD.*

*See for example*

*- Bidet-Ildei, Chavin & Coello, 2010 for the discriminability on PLD in scrambled masking dots*

*- Martel, Bidet-Ildei & Coello, 2011 for the sensitivity to the own actions.*

Response: In line with the reviewer’s suggestion, we now cite these relevant studies in the introduction:

*Furthermore, when asked to estimate the terminal location of a moving point-like arm that vanished after 60% of its movement, observers improved their performances when self-generated movements were presented (17).*

*Other results support this theory, showing for instance that producing a running activity briefly prior to the task improved participants’ perceptual judgements regarding the direction of a point-light runner (19),*

*I think that it misses in introduction something about the upside-down effect. It is surprising since this effect is specifically studied in Experiment 2.*

Response: We fully agree with the reviewer, and apologize for not citing this work earlier. We added a paragraph at the end of the introduction to emphasize this point:

*To that end, we relied on the classic method of presenting PLW upside-down, as it is known to specifically impair BM processing while conserving all local motion features as their upright counterparts (4,54,55). Changing orientation from upright to inverted impede spontaneous recognition of PLWs, making for instance more difficult to detect a camouflaged PLW within a mask (54).*

This effect is then mention in the discussion:

*Orientation manipulation of PLW is known to have a catastrophic effect on ability to extract information from PLWs: as in the case of static face representations, stimulus processing is adversely impacted when figures are presented upside down (4,12,54), even if observers are informed of seeing an inverted visual stimulus (54), a finding that has been taken as evidence that the neural mechanisms subserving BM processing are orientation-tuned.*

*METHODS:*

*-Are the same participants in Experiment 1 and 2?*

Response: No, participants from Experiment 1 and 2 were not the same. We now mention this important point in the method part:

*Participants in experiments 1 and 2 were not the same.*

*- Why the authors choose to describe both experiments and after to present the results? I find difficult to follow. I think that the manuscript could be clarified if the authors finished first the Experiment 1 and after proposing the Experiment 2. Moreover, in this configuration, they can more specified the specific objectives of each experiment and to add a specific discussion.*

Response: We would like to thank the reviewer for this advice. The reviewer is right to point that describing both experiment before the results made the reading of the manuscript more difficult. We have now moved the result section of Experiment 1 right after the description of the corresponding methods, and only then describe Experiment 2 and its results. However, we have kept the discussion of both experiments in the general discussion as we think it helps to keep in mind an overview of both experiments before hypothesizing possible mechanisms underlying the reported effects.

Please note that figure number changed with this new configuration: former Fig. 3 is now Fig. 2, and former Fig. 2 is now Fig. 3.

*DISCUSSION:*

*The authors explain their results with sensorial interactions but is it possible to imagine that their results could be due to more precise motor simulation of the PLW? Indeed, it would be logical that a tactile stimulation on the feet can reinforce the motor simulation of a walking motion and so improve the discriminability of PLW?*

Response: We fully agree with this point. We consider that tactile stimulation may up-regulate the vision-based embodiment of the observed walking action. Accordingly, it is possible that tactile stimulation improves the simulation of action in subjects’ own sensorimotor planning system. We apologize that this idea was not highlighted more clearly in the manuscript. We now emphasize this hypothesis in the text:

*In line with this role of motor simulation in BM perception, it is possible that tactile stimulations under the feet improve the observers’ motor simulation of the walking motion in their sensorimotor planning. This simulation, so reinforced by tactile congruent cues, could then improves the discriminability of PLW. Another study reports such possible reinforcement of body representation by multimodal perception:*

*Even this framework has difficulty to explain the results in Experiment 2 (the tactile effect with upside-down PLW), I think important to envisage this possibility. If the authors cut their study in two separate experiments, this hypothesis could be envisaged in the discussion of Experiment 1*

Response: The reviewer is right to point that results of Experiment 2 show that the effect isn’t specific to biological motion. This finding of Experiment 2 suggests indeed that synchronous tactile cues does not interact in the extraction of BM features, but in other motion features. We acknowledge that important point in the discussion.

Although we understand the reviewer’s point of view regarding the discussion format, we preferred discussing both experiments together to provide a more global view on the involved mechanisms. We are willing to change format should the reviewer and editor argue in this sense.
