# Supplementary material for: Somatosensory-visual effects in visual biological motion perception
Source: PLoS One. 2020 Jun 11;15(6):e0234026. doi: 10.1371/journal.pone.0234026 (PMC7289375; doi:10.1371/journal.pone.0234026)
Supplement: Attachment — Submitted filename: Response to Editor_26.11.19.docx [file pone.0234026.s002.docx]

Pierre Progin	November 26th, 2019

Laboratory of Cognitive Neuroscience

Swiss Federal Institute of Technology

Campus Biotech H4

Chemin des Mines 9

CH-1202 Genève

Switzerland

**RESPONSE TO EDITOR**

Dear Dr. Pavlova,

Thank you for providing us your valuable feedback on our manuscript titled “Somatosensory-visual effects in visual biological motion perception”. We are grateful for your insightful comments on our paper. We have been able to incorporate changes to reflect most of the suggestions you provided. We have highlighted these changes within the manuscript.

Here is a point-by-point response to your comments and concerns. Please, find below your comments repeated in italics and our responses inserted after each comment.

We look forward to hearing from you in due time regarding our submission and to respond to any further questions and comments you may have.

Sincerely,

Pierre Progin and co-authors

**EDITOR’S COMMENTS TO AUTHOR:**

**Comments to the Author:** *Two Reviewers who reviewed the previous version of your manuscript submitted their reports. I also had an attentive look at this version. I believe that you have address the following issues:*

*(i) Intro: You refer to work that supports co9nnection between perception and production of body motion. However, there are also reports that are not in aggrement with this view (e.g., from my own lab Pavlova et al., 2003, Brain). There are also several papers (e.g., Pavlova et al., 2017 Cerebral Cortex) with 9.4 fMRI and biological motion that you may wish to discuss.*

Response: We thank the Editor for pointing us to these relevant studies, which we now cite in the introduction:

“The planning system might be constitutional for the brain, as motor experience per se doesn’t seem to be necessary for BM perception. A study with young patients with periventricular leukomalacia revealed indeed that patients with early-life impaired motor ability showed the same sensitivity to BM that patients without motor disorder, leading to the assumption that a hard-wired network for both perception and production of BM might be inherent for the brain (28).”

“Furthermore a recent study using 9.4T functional MRI has shown that different neural circuits are activated for inverted or upright PLWs processing, with activation of left hemispheric anterior networks engaged in decision making and cognitive control for inverted BM, and activation of right hemispheric multiple networks in response to upright BM as compared with scarce activation to inverted displays (57).”

*(ii) REviewers did already draw your attention to unbalanced number of females and males in your experiments. Although you replied that binormial analysis does not show significant difference in number of female/male observers, you have to show that there were no gender difference in performance on your tasks to consider these groups homogeneous.*

Response: As requested, we computed the average performance across female and male observers. We found no difference for Experiment 1 (t(12.19) = 1.41, p = 0.18) and slightly better performance for males in Experiment 2 (77.1% vs. 74.4% t(10.05) = 2.48, p = 0.03). We now mention this possible issue in the discussion:

“Although our samples were not significantly unbalanced (see methods), the influence of gender on BM perception is worth considering. Indeed, we found no difference for Experiment 1 (t(12.19) = 1.41, p = 0.18) but slightly better performance for males in Experiment 2 (77.1% vs. 74.4% t(10.05) = 2.48, p = 0.03). This is in line with previous findings showing that female and male observers were found to judge differently whether a frontal PLW is facing toward or backward them (59), as well as emotional expressions (60). Future studies will be required to assess whether cross-modal effects on motion processing depend on gender. “

*(iii) VERY IMPORTANT: In Method section (Subjects) you are writing that several participants (4 in Exp. 1 and 3 in Exp. 2) had been excluded because of attentional problems. You wrote: (see below for more details'. Please explain the reasons in the text where you did mention exclusion, and indicate how many females and males had been excluded, and how many of them  entered your data analysis.*

Response: We apologize if exclusion criteria were not explained in sufficient details. They are specified under the procedure section:

“In addition, there was a ¼ chance in each trial that both the first and the second PLW were the reference stimulus with gait frequency of 0.77 cycle/sec (catch trial). The responses to those trials were excluded from the adaptive procedure. These catch trials were introduced to keep the task challenging and to maintain the attention of the subjects. If the correct response ratio for these catch trials was below 50%, meaning that more than half of the responses were false alarm, the participant was excluded from the analyses (4 subjects in Experiment1 and 3 subjects in Experiment 2).”

We now mention in the subject section that explanation are in the procedure part of the manuscript, and indicate the gender of the 7 excluded subjects:

“In Experiment 1, data from one male subject were excluded after that individual interrupted the experiment multiple times. Furthermore, 4 subjects (1 male and 3 female) in Experiment 1 and 3 subjects (2 male and 1 female) in Experiment 2 were excluded from analyses due to attention deficit during the task, i.e. more than half of the catch trials were performed as false alarm (see below procedure part for more details). Thus it remained 17 subjects (5 women) in Experiment 1 and 18 subjects (6 women) in Experiment 2 for the analysis.”

*(iv) You are writing that your participants were students. However, you did mention that one of your inclusion criteria  was age till 60 yrs. Is it not confusing? Please carefully address these issues in your revision.*

Response: We are sorry if this point is confusing. One of the inclusion criteria for our behavioral experiments is an age between 18 and 60 years. As the participants were recruited through printed advertisements on notice boards at various sites in the EPFL campus, they were actually all student at the EPFL, but it wasn’t formally a inclusion criteria. The mean age was 27.19 years (with a 34 yo oldest subject) in Experiment 1 and 24.04 years (with a 29 yo oldest subject) in Experiment 2.

We’ve now removed the sentence stating that they were students.
