# Supplementary material for: Somatosensory-visual effects in visual biological motion perception
Source: PLoS One. 2020 Jun 11;15(6):e0234026. doi: 10.1371/journal.pone.0234026 (PMC7289375; doi:10.1371/journal.pone.0234026)
Supplement: Attachment — Submitted filename: Response to Editor_06.01.20.docx [file pone.0234026.s003.docx]

Pierre Progin	January 6th, 2020

Laboratory of Cognitive Neuroscience

Swiss Federal Institute of Technology

Chemin des Mines 9

CH-1202 Genève

Switzerland

**RESPONSE TO EDITOR**

Dear Dr. Pavlova,

We would like to thank you for further assessing our manuscript, and apologize for the delayed response due to the holiday break.

Here is a response to your comments and concerns.

**Comments to the Authors:** *I now had a possibility to look at your reply. Thank you for your efforts. I must admit that there is a problem, which requires youir attention: you wrote that whereas there were no gender differences in Experiment 1 (please indicate whether you did check the data sets for normality of distribution, if the data is not normally distributed, yoiu are unable to use parametric statistics such as t-Student), in Experiment 2 the gender differences are significant (p < 0.03).[Please indicate whether one-tailed or two-tailed statistics is used]. In this latter case, you are unable to consider the whole group consisting of femakes and makles as homogenious. Instead, you have to proceed with the data of females and males separately. I am very sorry, but you really have to address these important issues. The other issue is exclusion of subjects: you have to explain why so many participants made false alarms. Were they anxious? Was the task extremely demanding?*

Response: We indeed had verified that our data was normally distributed with a Shapiro-Wilk normality test. This was the case both in experiment 1 (W = 0.96, p = 0.67) and in experiment 2 (W = 0.96, p = 0.56). Following standard guidelines, and because we had no a priori hypothesis concerning the influence of gender in our study, we used a two-tailed test to compare a posteriori performance between males and females.

Instead of proceeding with the data of females and males separately in experiment 2, we added this as a covariate in our analysis. We consider this to be a better approach, as it avoids losing statistical power as would be the case with data splitting. Doing so, the mixed-effects logistic regression yielded essentially similar results as the ones we reported originally. Most importantly, the interaction between stimulus intensity and synchrony remained significant (X² = 11.05, p < 0.001). We added this important point to the revised manuscript.

Finally, regarding subjects exclusion, we now report in the revised manuscript that some participants had to be excluded because of attention deficit during the task, likely due to the length of the experiment (180 repetitions of PLWs pairs in experiment 1 and 240 repetitions of PLWs pairs in experiment 2). To our knowledge, the task did not trigger anxiety, but was indeed quite demanding.

We thank you once again for your thorough editorial work, and hope that our study is now suitable for publication in PLOS ONE.

Best regards,

Pierre Progin, on behalf of all coauthors.
