# Supplementary material for: Somatosensory-visual effects in visual biological motion perception
Source: PLoS One. 2020 Jun 11;15(6):e0234026. doi: 10.1371/journal.pone.0234026 (PMC7289375; doi:10.1371/journal.pone.0234026)
Supplement: Attachment — Submitted filename: response to reviewers_12.05.20.docx [file pone.0234026.s004.docx]

Pierre Progin	Mai 12^th^, 2020

Laboratory of Cognitive Neuroscience

Swiss Federal Institute of Technology

Campus Biotech H4

Chemin des Mines 9

CH-1202 Genève

Switzerland

**RESPONSE TO REVIEWERS**

Dear Dr. Pavlova,

Thank you for providing us your valuable feedback on our manuscript titled “Somatosensory-visual effects in visual biological motion perception”. We appreciate the time and effort that you and the reviewer have dedicated to our manuscript and are grateful to the reviewer for her/his insightful comments on our paper. We have been able to incorporate changes to reflect most of the suggestions provided by the reviewers. We have highlighted the changes within the manuscript.

Here is a point-by-point response to the reviewer’s comments and concerns. Please, find below the reviewer’s comments and our responses inserted after each comment.

We thank you once again for your thorough editorial work, and hope that our study is now suitable for publication in PLOS ONE.

Best regards,

Pierre Progin, on behalf of all coauthors.

**EDITOR’S COMMENTS TO AUTHOR:**

**Comments to the Author:** We received feedback from Reviewer 3 (you can find her/his comments below). I believe that two issues are of importance:

1. Stricktly speaking your groups are imbalannced in regard to gender. Experiment 1 contained 8 female and 22 male subjects, Experiment 2 contains 21 male and 7 femnale participants. Binomial test is of no help here. If you have such an imbalanced design, your statisticakl outcome can be affected heavily.

2. The difference between female and male participanrts WAS significant. Significant difference can't be negligible. Please address these issues in your work.

Response: We now make the following statement as an important limitation in the discussion:

“[...] our sample of participants was not balanced for gender. Only 8 women of 22 subjects participated in Experiment 1 and 7 women of 21 subjects in Experiment 2. Previous studies have shown differences in BM perception depending on gender, showing for instance that female and male observers were found to judge differently whether a frontal PLW is facing toward or backward them (39), as well as emotional expressions (40). Of note, post-hoc analysis revealed better performance in PLW discrimination in male observers in Experiment 2 (77.1% vs. 74.4% t(10.05) = 2.48, p = 0.03), but not in Experiment 1 (t(12.19) = 1.41, p = 0.18). A supplementary binomial mixed-effects regression including gender as a covariate in Experiment 2 revealed that the interaction between intensity and synchrony remained significant (X² = 11.41, p < 0.001), and was not modulated by gender (X² = 0.66, p = 0.41). Future studies will be required to assess whether this effect is anecdotal or whether cross-modal effects on motion processing depends on gender”.

**REVIEWER’S COMMENTS TO AUTHOR:**

**Comments from Reviewer #3:** I have a few concerns that follow. To sum them up [1] we need to pay attention to variables operationalization and correspondent stimuli effectiveness for cross modality stimulation (mirror neuron like integration vs higher order cognitive-attentional convergent information about a variable); [2] it is not clear the gender effect; we should [3] pay attention to minor language problems. I won't use locutions as "in my opinion" etc. because tautological. I apologize for any unpoliteness that should be perceived.

[1] The effectiveness of the asynchronous condition depends on the following fact. The stimulus in its simplest representation can be described in terms of frequency and phase. The asynchronous condition has to be both different in "frequency" and "phase", otherwise we cannot discard the simplest interpretation that subjects use a low level property of perception (frequency) to give their responses. I try to be clearer.

The stimulus PLW can be described in terms of frequency of an event ("the [perceived] foot touches the ground"): the higher the frequency, the higher the psychophysical variable we want to measure (the [perceived] velocity of the [perceived] walker). In the synchronous condition there is another stimulus (processed in another perceptual modality) which matches with plw both in phase and in frequency (or at least - please clarify this point - with a frequency which is an integer multiple or divisor of the frequency of the plw event).

For example:

SAME PHASE - SAME FREQUENCY

time plw foot stimulus

1 1 1

2 0 0

3 0 0

4 1 1

5 0 0

6 0 0

7 1 1

8 0 0

9 0 0

...

DIFFERENT PHASE - SAME FREQUENCY

time plw foot stimulus

1 1 0

2 0 1

3 0 0

4 0 0

5 1 0

6 0 1

7 0 0

8 0 0

9 1 0

10 0 1

11 0 0

12 0 0

...

SAME PHASE - DIFFERENT FREQUENCY (but integer multiple: frequency foot touch = 2 * frequency plw)

time plw foot stimulus

1 1 1

2 0 0

3 0 1

4 0 0

5 1 1

6 0 0

7 0 1

8 0 0

9 1 1

10 0 0

11 0 1

12 0 0

...
ecc.

The point is: the asynchronous condition should be different both in phase and in frequency with no frequency at all preferably (this can be set giving a random order of the "1's" in the event representation, but maintaining that the total number of "ones" is the same for both stimuli. In analytic terms the two [continuous] event (y) - time (x) functions should have the same integral but one should be a sine(x+shift) and the other some random thing with the same area

Response: We thank the reviewer for noting this important aspect and we apologize if our description of the tactile stimuli were not clear enough in the original manuscript.

Actually, in the synchronous stimulation, the visual stimuli and the tactile stimuli had the same frequency and phase. The visual stimuli consisted of two stimuli, PLW 1 and PLW 2, separated by 1500 ms intervals. The gait frequency was 0.77 cycles per second for PLW 1 and (0.77 + Δf cycle/sec) for PLW 2 according to the participants performance (staircase adaptative procedure). PLW 1 or PLW 2 could be presented with equal probability as the first visual stimulus for each trial. The duration of the tactile stimuli was adapted depending on the duration of the visual stimulus (i. e. if the PLW walked faster, the tactile stimulation was shorter) and corresponded to 20% of the duration of the total PLW gait. Indeed, for a full phase of a walking cycle, Foot 1 was stimulated between 10-30% of the visual stimuli cycle and Foot 2 between 60-80% of this cycle. If the cycle was longer/shorter, these stimulation windows were adjusted accordingly.

To complete your example, we can schematize this as (in row “PLW” **1** indicated that the feet of the PLW touch the ground, in rows “Foot” **1** indicated that tactile stimulations are activated):

SAME PHASE - SAME FREQUENCY

| Time | PLW1 | Foot1 | Foot2 |
| --- | --- | --- | --- |
| 1 | 0 | 0 | 0 |
| 2 | **1** | **1** | 0 |
| 3 | **1** | **1** | 0 |
| 4 | 0 | 0 | 0 |
| 5 | 0 | 0 | 0 |
| 6 | 0 | 0 | 0 |
| 7 | **1** | 0 | **1** |
| 8 | **1** | 0 | **1** |
| 9 | 0 | 0 | 0 |
| 10 | 0 | 0 | 0 |

Interval 1500 ms

| Time | PLW2 | Foot1 | Foot2 |
| --- | --- | --- | --- |
| 11 | 0 | 0 | 0 |
| 12 | **1** | **1** | 0 |
| 13 | **1** | **1** | 0 |
| 14 | 0 | 0 | 0 |
| 15 | 0 | 0 | 0 |
| 16 | 0 | 0 | 0 |
| 17 | **1** | 0 | **1** |
| 18 | **1** | 0 | **1** |
| 19 | 0 | 0 | 0 |
| 20 | 0 | 0 | 0 |

In the asynchronous condition, both frequency and phases were different for the tactile and visual stimuli. The duration of the tactile stimulation was the same as in the synchronous condition (corresponding of 20% of the total PLW gait cycle), but for each foot the phase was randomly selected. For example, this could result in Foot 1 being stimulated from 0-20% of the cycle and Foot 2 from 15-35%. The phase was randomized for each foot separately, i. e. it could also be that Foot 1 and Foot 2 were stimulated at the same time.

For example, it could be like this:

DIFFERENT PHASE – DIFFERENT FREQUENCY

| Time | PLW1 | Foot1 | Foot2 |
| --- | --- | --- | --- |
| 1 | 0 | **1** | 0 |
| 2 | **1** | **1** | **1** |
| 3 | **1** | 0 | **1** |
| 4 | 0 | 0 | 0 |
| 5 | 0 | 0 | 0 |
| 6 | 0 | 0 | 0 |
| 7 | **1** | 0 | 0 |
| 8 | **1** | 0 | 0 |
| 9 | 0 | 0 | 0 |
| 10 | 0 | 0 | 0 |

Interval 1500 ms

| Time | PLW2 | Foot1 | Foot2 |
| --- | --- | --- | --- |
| 11 | 0 | 0 | 0 |
| 12 | **1** | 0 | 0 |
| 13 | **1** | 0 | 0 |
| 14 | 0 | **1** | 0 |
| 15 | 0 | **1** | 0 |
| 16 | 0 | 0 | 0 |
| 17 | **1** | 0 | 0 |
| 18 | **1** | 0 | **1** |
| 19 | 0 | 0 | **1** |
| 20 | 0 | 0 | 0 |

We added these important methodological details in the Method section:

“The duration of the tactile stimuli was adapted depending on the duration of the visual stimulus (i.e. when PLWs walked faster, tactile stimulations were shorter) and corresponded to 20% of the duration of the total PLW gait.

In the ‘synchronous’ conditions of Experiment 1 and 2, stimulation onset of the laterally matched body part was timed to coincide with the point at which the PLW ‘ankle’ dot reached its lowest value on the y-axis. Indeed, for a full phase of PLW cycle, Foot 1 was stimulated between 10-30% of the visual stimuli cycle and Foot 2 between 60-80% of this cycle.”

“In the ‘asynchronous’ condition of Experiment 2, tactile stimulation onset was randomized during the visual stimulus. Both frequency and phase were different for the visual stimuli and the tactile stimuli. The duration of the tactile stimulation was the same as in the synchronous condition (corresponding of 20% of the total PLW gait cycle), but for each foot the phase was randomly selected. For example, this could result in Foot 1 being stimulated from 0-20% of the cycle and Foot 2 from 15-35%. The phase was randomized for each foot separately, i. e. it could also be that Foot 1 and Foot 2 were stimulated at the same time.

Please add some further consideration about the effectiveness of your "somatosensory drum effect" (some tips about implicit questions you could ask again yourself):

- how can I distinguish the pure "rythm" attentive effect from the "cross modality - mirror neuron like one"? Is my Asynchronous condition enough as it stands? etc.

Response: For Experiment 1, we consider unlikely that the effect comes from a “drum” or “rhythm” effect, as it was not present when the same tactile stimulation was delivered on the forearms. The experimental tactile condition (ET) and control tactile condition (CT) contained indeed the same visual and tactile information about the speed of the PLW.

We now add this to the Discussion:

“As the frequency and the phase of both visual and tactile stimuli were the same in this experiment, the tactile input itself (through its frequency and its duration) could have conveyed additional information about the speed of the PLW. It is then possible that the improvement in BM discriminability was only due to this additional information. But the finding that the same tactile stimuli improved BM discrimination only when they were delivered under the feet makes this explanation unlikely. There was indeed no difference in BM discrimination when no tactile stimulation occured (V) and when tactile stimulation was applied on the forearms (CT).”

For Experiment 2, as the frequency and the phase are different for the visual and tactile stimuli in the asynchronous condition, it is possible that the better speed discrimination in the synchronous condition comes from such “rhythm” matching between the two sensory inputs.

We now discuss this possibility in the Discussion:

“However, as the frequency and the phase are different for the visual and tactile stimuli in the asynchronous condition, we cannot exclude, based only on Experiment 2, that the improvement in BM discriminability in the synchronous condition comes from additional information about the speed of the PLW conveyed by the tactile stimulation itself.”

- How long does each PLW stimulus last?

Response: We used the monitor refresh rate as the reference (100Hz) and each frame showed a different dot pattern. The gait frequency of 0.77 cycles per second was used as the base gate frequency, meaning that 129 frames were shown for a full PLW cycle, lasting 1298 ms. An interval of 1500 ms separated the presentation of this reference PLW to a second PLW with a slightly faster gait frequency (0.77 + Δf cycle/sec) according to the participants performance (staircase adaptative procedure). For instance, if during the previous trials the discrimination was incorrect with a Δf of 0.2 cycle/sec, then the second walker would have 100/(0.77+0.2) = 103 frames for a full cycle, lasting 1030 ms. Then the total duration varied for each trial, with a maximum of 4096 ms (1298 + 1500 + 1298 ms).

- is it necessary to refer to the [complex and not universally accepted] theory of mirror neurons or the results can be [easily] explained by other (simpler) mechanisms?

In other words if we cannot exclude a simpler process of converging information (different stimuli of which frequencies correlates and help the performance which has a "computation time" compatible with higher order processes), the discussion, say, of rows 461 - 499 has to be "mitigated". Could some further considerations regarding the comparison of results for forearm vs foot stimulation help?

Response: Following the Reviewer’s suggestion, we have left the reference to mirror neurons, which was not the aim of this study, and now also propose alternative hypotheses regarding the difference of results for forearm and feet stimulation in the Discussion section (i.e., difference in sensitiveness between the two body parts, higher relevance of the feet for an embodied representation of walking actions).

Should this considerations refer also to possible different sensitiveness of those areas (example rows 311-312)?

Response: The sensitivity of the tactile simulation on the arm was tested in a pilot study. Moreover, participants reported strong tactile sensations during the task. Accordingly, we argue that it is unlikely that performance difference in Experiment 1 are due to a difference in sensitiveness between feet and forearms, although we cannot exclude this. We now have mentioned this limitation in the revised Discussion:

“Secondly, we cannot exclude the possibility that differences in tactile perception between the forearms and the feet may have resulted from difference in sensitiveness between these two body parts. We hypothesized during the study conception that the feet would be more relevant than forearms regarding walking actions, and therefore predicted that performance would be better with tactile stimulation on this site than elsewhere on the body.”

[2] balanced design are always preferable; your design is not regarding gender and it's unbalanced from the very beginning (it is not only a matter of subjects exclusion for data or inclusion problem). I would not mention at all gender variable effect.

Response: We share the reviewer’s opinion, but decided to follow the editor’s suggestion and report post-hoc effects on gender (see above).

If you are somehow obliged to, I'd rather be more explicit about the reasons that make compatible the following statements:

rows 415-417: gender variable has a main effect in experiment 2 and does not have it in experiment 1.

rows: 420-422: the effect of intensity x synchrony remain significant on the dependent variable (the fuzzy-step|sygmoid|error function like decision function) if in the model you add gender variable explicitly.

For what I can understand: on one hand, on its own, if you use t-test, gender has no effect in experiment 1 but has an effect in experiment 2; on the other hand, it does not "significantly modulate" the combined effect of stimulus (plw) intensity and synchrony (with somatosensory stimulation). Those results are compatible without the need of giving any further information on experiment 1; on experiment 2 we are lead to think that (a) the "effect size" of intensity x synchrony is high enough to "cover" the one of gender on its own or [inclusive] (b) that the interaction gender x intensity x synchrony is "small" if significant. If this representation of what you mean in rows 414-424 is correct, please give some statistical effect size measure and be more analytic in its explanation, above all whether if the above a, b or both are true.

Response: Following the reviewer’s suggestion, we conducted a supplementary binomial mixed-effects regression including gender as a covariate of (no) interest in Experiment 2. We are pleased to report that the interaction between intensity and synchrony remained significant (X² = 11.41, p < 0.001), and was not modulated by gender (X² = 0.66, p = 0.41).

In layman terms you should represent you results in order to explain both (1) why gender has effect in experiment 2 and not in experiment 1 and (2) why gender does not affect the intensity x synchrony effect.

Maybe it could be helpful to "let us see" the model. I guess it is something like this:

to each discrete independent variable V_k with number of levels N_k we associate N_k variables X_h with values 0 or 1.

to each numerical/continuous independent variable W_i we associate a numerical variable X_i with the same domain.

So from

[subject, intensity, synchrony, gender]

we obtain

x_1, ..., x_n, with n = number_of_subjects

x_n+1 =intensity

x_n+2 = asynchronous_stimulation

x_n+3 = synchronous_stimulation

x_n+4 = female

x_n+5 = male

and the model WITHOUT interaction is

y = sum(a_j * x_j) + error

For the one with interaction you add more variable such as

x_n+6 = (x_n+1)*(x_n+2 == 1) that is intensity for asynchronous stimulation and so on.

If my rough resume is somehow correct, it should be the case that

y = ... + a*(intensity_x_synchrony) + b*(intensity_x_synchrony_x_gender) + c*(gender) + ...

gives

a significant and strong

b significant and small (or not significant)

c significant (for experiment 2) and small (comparing to a)

am I correct? Nevertheless I suggest you try to find a way to make it clearer

Response: The reviewer is correct. We provide below the table corresponding to the analysis of Deviance (Type II Wald chi square tests) of the model: resp ~ intensity* synchrony * orientation * sex

[3] Miscellanea and minor concerns

Row 244: "...was randomized between each body part ...": check for "between" as it's used for a set of two elements, so it should be "among", because I guess that the body parts are more than 2. Moreover I think you may want something like "... randomized across ... " or "... randomly sampled among all body parts ...". However, if by "body parts" you do not mean those related to moving dots but those of the experimental subject, please be more clear about why you should randomize "left and right" stimulation (I guess because you have only one tool). Nevertheless please check this part with a native speaker.

Response: We would like to thank the reviewer for this important comment. We meant *among* each body parts. We now give more details about the tactile stimuli in the Apparatus and Stimuli section:

“In the ‘asynchronous’ condition of Experiment 2, tactile stimulation onset was randomized during the visual stimulus. Both frequency and phase were different for the visual stimuli and the tactile stimuli. The duration of the tactile stimulation was the same as in the synchronous condition (corresponding of 20% of the total PLW gait cycle), but for each foot the phase was randomly selected. For example, this could result in Foot 1 being stimulated from 0-20% of the cycle and Foot 2 from 15-35%. The phase was randomized for each foot separately, i. e. it could also be that Foot 1 and Foot 2 were stimulated at the same time.”

Row 414: "Although ... NOT ..., the influence is worth considering". I cannot understand this sentence. "Although B, A" means that "A" usually implies "not-B"; in other words "A although B" should be something like "A even if not-B".

A="the influence of gender on BM perception is worth considering"

B="our samples were not significantly unbalanced for gender"="our samples were balanced enough for gender"

so to maintain an intuitive semantics it should be "A because B". Please clarify this point.

Response: The reviewer is right, this sentence was incoherent. Actually there was a mistake in the first part of the sentence, it should have been: “although our samples were not significantly *balanced* for gender […]” and not “[…] *unbalanced* […]”. We don’t use this sentence anymore in the manuscript.

Row 450: "STS, with its central [...] actions (see [...]) has been implicated [...]" should be

"STS, with its central [...] actions (see [...]), has been implicated [...]" (the comma is missing). Please double check the paper because there are other punctuation errors.

Response: We thank the reviewer for these observations. We have double checked the revised manuscript for punctuation errors.
